# Facile Synthesis and Self-Assembly of Amphiphilic Polyether-Octafunctionalized Polyhedral Oligomeric Silsesquioxane via Thiol-Ene Click Reaction

**DOI:** 10.3390/polym9070251

**Published:** 2017-06-28

**Authors:** Yong Xia, Sha Ding, Yuejun Liu, Zhengjian Qi

**Affiliations:** 1 Key Laboratory of Advanced Packaging Materials and Technology of Hunan Province, Hunan University of Technology, Zhuzhou 412007, China; 421298314@163.com (S.D.); yjliu_2005@126.com (Y.L.); 2 College of Chemistry and Chemical Engineering, Southeast University, Nanjing 211189, China; qizhengjian506@163.com

**Keywords:** amphiphilic, thiol-ene chick chemistry, CMC, thermo-sensitive

## Abstract

We demonstrated here a facile and efficient synthesis of polyhedral oligomeric silsesquioxane-based amphiphilic polymer by thiol-ene click chemistry. The properties of polyhedral oligomeric silsesquioxane (POSS)–PEG amphiphilic polymers were studied in detail by a combination of ^1^H NMR, ^13^C NMR, ^29^Si NMR FT-IR, GPC, and TG analysis. The newly-designed thiol-ene protocol obtains only anti-Markovnikov addition POSS-based amphiphilic polymers when compared with platinum-catalysed hydrosilylation method. The critical micelle concentration (CMC) of the resulting polymers are in the range of 0.011 to 0.050 mg/mL, and dynamic light scattering (DLS) results revealed that the obtained amphiphilic polymers can self-assemble into nanoparticles in aqueous solutions with a bimodal (two peaks) distribution. Furthermore, the specific polymer showed obvious thermo-sensitive behaviour at 45.5 °C.

## 1. Introduction

In recent years, organic/inorganic hybrid nanomaterials have aroused widespread interest. Since they are combined with the traditional organic polymers’ easy workability, toughness, and thermal stability and oxidation resistance properties of inorganic compounds, this kind of materials play a key role in the development of high performance and high functional materials [1,2,3,4]. At present, there are many different types of nanocomposites, such as three-dimensional sol-gel materials [5,6,7,8,9], ceramic polymer [10,11,12], organic/inorganic polymer blend [13,14,15], and polyhedral oligomeric silsesquioxane (POSS) nanocomposites [16,17,18,19,20,21]. POSS-based polymer is a kind of nanoscale silicate functional material with cage-like segments directly bound to the polymer chains. Such newly-developed nanocomposite materials show synergistic properties of organic/inorganic materials, and present some new characteristics [22,23].

POSS has a nanoscale silicon core and eight organic functional groups on the surface, with sizes from 0.7 to 1.5 nm. The three-dimensional nanostructures of POSS can be used to build all types of hybrid materials with specific performance and controllable nanostructures [24]. Attracted by the fascinating vistas, POSS has potential applications in high-performance materials and biomedical fields [25,26,27,28], but its low water-solubility is the main impediment for real-world applications. Grafting hydrophilic groups on POSS is one of the effective methods to achieve water-soluble materials [29,30]. As is known to all, polyethylene glycol (PEO) is a water-soluble polymer with excellent properties, such as non-toxicity, lubricity, and biocompatibility. PEO is widely applied in biotechnology, in addition, it is a type of very important solid polymer electrolyte in lithium ion batteries because of its ability of dissolve the lithium ion [31]. Kim et al. [32] reported the synthesis and characterization of amphiphilic telechelics incorporating polyhedral oligosilsesquioxane (POSS–PEG). They obtained amphiphilic telechelic polymers with different POSS content by changing the PEG molecular weight, and studied the effect of POSS content on the crystallization behaviour of PEG. The results showed that different morphology and thermodynamic properties of the amphiphilic telechelic polymers could be obtained through controlling the ratio of hydrophilic PEG homopolymer and hydrophobic POSS macromolecular monomer. Subsequently, Kim et al. [33,34] systematically studied the polymer morphology, microstructure and rheological properties of POSS–PEG, and the impacts of structure and solvent polarity on bonding behavior in dilute solution using dilute solution viscosity method. Maitra et al. [35] synthesized a series of ethylene oxide oligomers functional silsesquioxane, reveals how the POSS silicon surface affect thermal behavior of ethylene oxide oligomers, *T*_g_ increases with the increase of the length of ethylene oxide chain, and inhibiting crystallization.

Although there have been many reports about the synthesis and application of polyethylene oxide functionalized silsesquioxane [36,37,38,39,40,41,42,43], but the preparation methods generally involve the Pt-catalyzed hydrosilylation between the terminal Si–H groups on the POSS cube with an unsaturated carbon double bond. The traditional method possesses some disadvantages: this method requires noble metal platinum, which is very expensive. It is difficult to get rid of the residues in purification process, which not only affects the product performance, but also limits its application in the field of biological medicine. This reaction generally requires strict water-free and oxygen-free conditions. The hydrosilylation reaction usually results in a mixture of Markovnikov and anti-Markovnikov addition products [44], which might complicate the structure-activity relationship of the amphiphilic copolymer. In addition, it is very difficult to obtain the high hydrophilic eight substitution product due to the limit of reaction efficiency and the influence of steric hindrance. Li et al. [45] successfully synthesize the eight functional POSS–PEG polymer using the copper-catalyzed alkyne-azide cycloaddition, which has faster reaction rate and higher yield. Nevertheless, the residues of heavy metal copper catalyst still have a certain influence on its application in the biomedical field. Therefore, a series of eight polyethylene oxide functionalized POSS amphiphilic polymers are synthesized through the metal-free thiol-ene click reaction, theirs self-assembly behaviour in aqueous solution, thermal stability, and thermo-responsive are systematically studied.

## 2. Experimental Section

### 2.1. Materials 

Allyl-terminated polyether **A** (1000 g/mol, EO/PO = 70/30), **B** (1200 g/mol, EO/PO = 85/15), and **C** (2000 g/mol, EO/PO = 40/60) were provided by Jiangsu Maysta Chemical Co. Ltd. (Nanjing, China) 2,2-dimethoxy-2-phenylacetophenone (DMPA) was purchased from Aladdin (Shanghai, China) and used as received. Mercaptopropyltrimethoxysilane (MPT) and tetramethylammonium hydroxide ((CH_3_)_4_NOH) were purchased from J and K Chemical (Shanghai, China) and used without any further purification. Tetrahydrofuran (THF), diethyl ether, acetone, and concentrated hydrochloric acid were of analytical grade. In this work, the thiol-ene reaction was carried out under ambient conditions, the other reactions were carried out under a dry nitrogen atmosphere. The photo-induced reactions was carried out by a UV lamp (20 mW·cm^−2^, λ = 365 nm; LP-40A; LUYOR Corporation, Shanghai, China).

### 2.2. Synthesis of Octamercaptopropyl-POSS (POSS–SH)

POSS–SH is prepared according to our previous work [46]. The resulting viscous solution was dissolved in CH_2_Cl_2_ and then washed three times with H_2_O. The CH_2_Cl_2_ phase was dried with anhydrous Na_2_SO_4_ and concentrated to obtain POSS–SH in 76% yield. IR (KBr; *ν*, cm^−1^): 2929 (m), 2555 (w), 2849 (m), 1401 (m), 1122 (vs), 1032 (s), 562 (m), 472 (m). ^1^H NMR (300 MHz, CDCl_3_; δ, ppm): 0.76 (double, Si–CH_2_–), 1.71 (s,–CH_2_–), 2.56 (s, –CH_2_–S), 1.37 (br, –SH). ^13^C NMR (300 MHz, CDCl_3_; δ, ppm): 13.46 (Si–CH_2_–), 29.87 (Si–CH_2_–CH_2_–), 30.16 (–CH_2_–SH). ^29^Si NMR (400 MHz, CDCl_3_; δ, ppm): −67.20. GPC-result, *M*_n_: 1005 g/mol, PDI: 1.02.

### 2.3. Synthesis of Polyether-Octafunctionalized POSS (POSS–PEG)

**Scheme 1:**
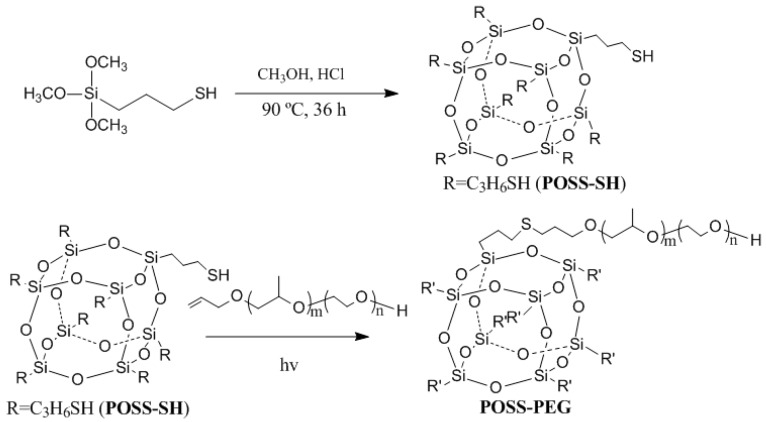
The synthesis of POSS–PEG polymer.

For the purpose of discussions, the POSS–PEG amphiphilic polymers containing different allyl-terminated polyethers are defined as **P_1_**, **P_2_**, and **P_3_**, respectively. The synthetic route of POSS–PEG amphiphilic polymer is shown in Scheme 1. The **P_1_** was synthesized by thiol-ene click chemistry as follows: 0.5 g octamercaptopropyl-POSS (POSS–SH), 4.4 g allyl-terminated polyether **A** and 0.08 g DMPA were dissolved by 8 mL dry THF solvent. The system was stirred gently for 30 min under UV light irradiation, then placed in a dialysis bag (cutoff *M*_n_: 3.5 kDa) and dialyzed against water for three days to get rid of residual polyether, replacing dialysate every 4 h and, finally, obtaining the target product by drying the inside mixture. Yield: 98%, ^1^H NMR (300 MHz, CDCl_3_, ppm): δ = 0.71 (m, SiCH_2_–), 1.08–1.13 (d, –CH_3_), 1.64 (t, –SiCH_2_CH_2_–), 1.81 (m, –SCH_2_CH_2_–), 2.51 (t, –CH_2_SCH_2_–), 3.27–3.85 (m, –OCH_2_CH_2_O–, –OCH_2_CH(CH_3_)O–). GPC-result, *M*_n_: 13510 g/mol, PDI: 1.15.

The synthetic methods of **P_2_** (polyether: *M*_n_ = 1200 g/mol, EO/PO = 85/15) and **P_3_** (polyether: *M*_n_ = 2000 g/mol, EO/PO = 40/60) are identical to **P_1_**.

### 2.4. Characterizations

FT-IR was carried out using a Bruker TENSOR27 (Ettlingen, Germany) with the KBr pellet technique within the 4000 to 400 cm^−1^ region. ^1^H NMR was carried out on a Bruker Avance (Billerica, MA, USA) 300 and 400 MHz at 25 °C. The solvent was deuterated chloroform (CDCl_3_) without the interior label. The measurements of GPC were recorded on PL-GPC220 (Agilent, Palo alto, CA, USA) with THF as the eluent (1.0 mL/min) at 40 °C. TG analysis was performed with a STARe System (Mettler Toledo, Columbus, OH, USA). The heating rate was 20 °C/min under N_2_. TEM was recorded on a JEOL 2100 high-resolution TEM (JEOL Ltd., Tokyo, Japan) and Philips (CM 200, 80 kV, Amsterdam, The Netherlands). 

### 2.5. Critical Micelle Concentration (CMC) Measurements

The CMC of the copolymers in ultrapure water was determined by fluorescence spectroscopy using pyrene as a hydrophobic fluorescent probe [47,48]. Briefly, a pyrene solution (6.2 × 10^−6^ M in acetone, 1 mL) was placed in different vials, and the solvent was evaporated. Then, different concentrations of copolymer (10 mL) were added to those vials, respectively. Thus, the final concentration of pyrene was 6.2 × 10^−7^ M and the concentrations of polymer micelles were from 1.0 × 10^−4^ to 1.0 g/L. Fluorescence measurements were carried out on a FluoroLog 3-TCSPC (Horiba Jobin Yvon Inc., Edison, NJ, USA) with a xenon light source (UXL-150S, Ushio, Japan). The slit widths of emission and excitation were 2 nm. The excitation spectra were recorded from 290 to 360 nm with the emission 373 nm. The emission spectra values *I*_338_ and *I*_335_, at 338 and 335 nm, respectively, were used for subsequent calculations. The CMC was determined by plotting the *I*_338_/*I*_335_ ratio against the polymer concentration. The CMC was taken as the intersection of regression lines calculated from the linear portions of the plot.

### 2.6. Dynamic Light Scattering (DLS) 

The DLS measurements were performed by a Brookhaven BI-200SM instrument (Holtsville, NY, USA) equipped with a 4 mW He-Ne laser (λ = 532 nm) at an angle of 90°. The micelle copolymer solutions, with concentration 1 g/L, were prepared by direct dissolution of copolymers in ultrapure water. These samples, which were standing for 24 h before measurement, were filtered through 0.45 μm PTFE microfilters. The results of the test were recorded using the intensity-weighted distribution of particle sizes.

## 3. Results and Discussion 

### 3.1. Characterization of Octamercaptopropyl-POSS (POSS–SH)

The absorption bands of Si–O–Si asymmetric stretching vibration are situated at 1109 and 1037 cm^−1^, showing in Figure S1. The large peak area may indicate that the obtained POSS–SH is composed primarily of Si–O–Si structure. The bands at 562 and 469 cm^−1^ are ascribed to the deformation vibration of POSS skeletal, and the stretching vibration of the bridge between caged silicon core and organic ligand Si–C is located at 693 cm^−1^. The bands at 2930, 2819, and 1407 cm^−1^ are assigned to the stretching and bending vibrations of –CH, while those at 1261 and 802 cm^−1^ are attributed to C–C stretching vibration. It is even more exciting to clearly see the characteristic absorption of –SH stretching vibration, which is located at 2555 cm^−1^.

^1^H NMR spectrum of POSS–SH (Figure S2) illustrates four strong resonance signals corresponding to different H atoms in its structure. The chemical shifts of these protons are assigned at 0.76 ppm (double, SiCH_2_–), 1.71 ppm (s, –CH_2_–), 2.56 ppm (s, –CH_2_S), and 1.37 ppm (br, –SH), which agree well with a, b, c and d groups on the side chains of the POSS–SH framework. ^13^C NMR spectrum (Figure S3) shows that the chemical shift at 13.46 ppm is assigned to the –CH_2_ group (carbon a) directly bound to the Si atom, while the others at 29.87 and 30.16 ppm are attributed to carbon b and c of the mercaptopropyl group, respectively. On the other hand, the ^29^Si NMR spectrum (Figure S3) reveals a sharp peak with a chemical shift at 67.20 ppm, ascribing to the Si atom of the POSS–SH. The results suggest that the cage-like Si–O–Si structure is well formed by the hydrolytic condensation of MTP and mercaptopropyl groups are symmetrically distributed on the cage-like POSS skeletal.

### 3.2. Characterization of Amphiphilic Polymers

Amphiphilic POSS–PEG polymers were synthesized by thiol-ene click chemistry with polyhedral oligomeric silsesquioxane and allylic polyether as starting materials.

**Figure 1:**
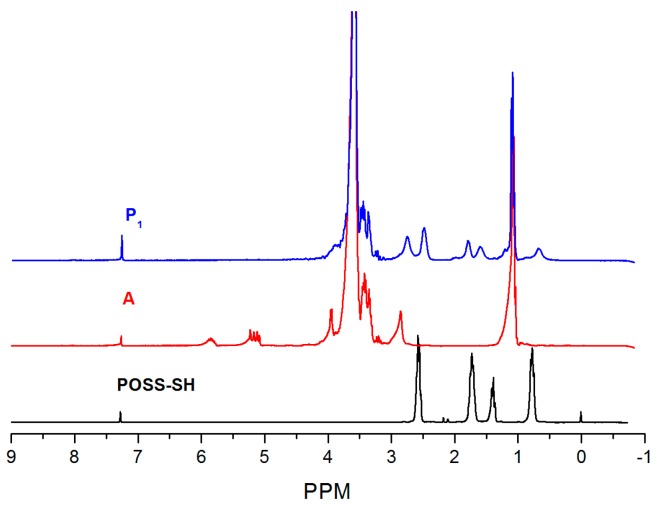
^1^H NMR spectra of **P_1._**

In ^1^H NMR (Figure 1) of **P_1_**, all of the proton signal belonging to the polyether fragment and the POSS–SH fragment can be observed. The disappearance of S–H signal at 1.37 ppm and vinyl proton signal at 5.11 to 5.88 ppm confirmed the formation of **P_1_**. As previously reported, the thiol-ene reaction (Si–Vi and –SH) may give either of two possible products (Markovnikov and anti-Markovnikov) [49,50,51]. The anti-Markovnikov addition product is dominant. However, the result of ^1^H NMR indicates that there seem to be only anti-Markovnikov addition product in the current system. The number of epoxy propane (PO) unit in each polyether molecule was calculated by ^1^H NMR of allyl polyether, then the number of polyether chains grafted on POSS could be determined by the integral area ratio of methyl at 1.08–1.13 ppm and methylene at 0.71 ppm. The calculated results indicate that nearly eight polyether chains were grafted on each POSS, achieving entirely replace. However, due to the steric effect of high molecular weight polyether, amphiphilic POSS–PEG polymers synthesized by the hydrosilation method were difficult to be fully substituted. For example, Mya et al. [52] prepared POSS–PEG polymers by the hydrosilation, in which each polymer had only six polyether chains grafted onto silsesquioxane. The ^1^H NMR of **P_2_** and **P_3_** were similar to the result of **P_1_**.

**Figure 2:**
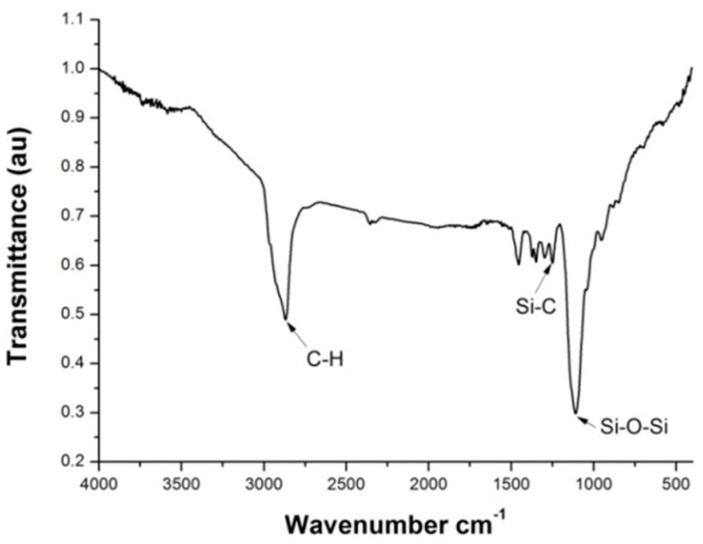
The FT-IR spectra of **P_1._**

The IR spectrum of **P_1_** (Figure 2) clearly showed the symmetrical stretching vibration of Si–O–Si at 1111 cm^−1^, corresponding to the cage structure of silsesquioxane. The stretching vibration and bending vibration of Si–C were located at 1252 and 954 cm^−1^, respectively. Absorption bands at 954 and 843 cm^−1^ were characteristic peaks for Si–C groups on its outside surface and the crystallization phases of polyether. Characteristic peaks of thiol at 2555 cm^−1^, and those peaks of olefin C–H and C=C disappeared, which suggested the completion of the click reaction.

**Figure 3:**
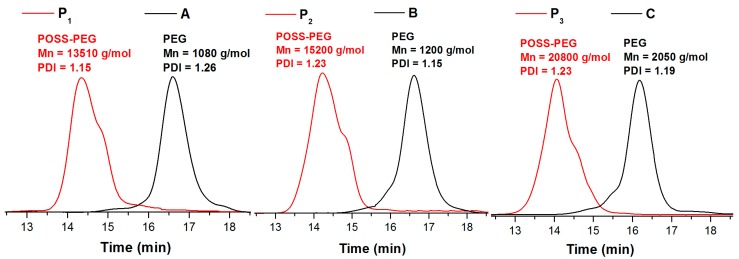
The GPC profiles of allyl-polyether and octa-substituted POSS polymers.

Further verification of the successful synthesis was obtained from GPC analyses. Figure 3 shows representative GPC profiles of the Allyl-terminated polyether **A**, **B**, and **C** and the subsequent polymers (**P_1_**, **P_2_** and **P_3_**). A significant shift in the GPC traces (to a higher molecular weight) of the PEG and monomodal molecular weight distributions of the polymers indicates a successful click chemistry producing well-defined polymers. Furthermore, all of the three polymers have narrow molecular weight distribution, which are 1.15, 1.23, and 1.23, respectively. The molecular weight of **P_1_**, **P_2_**, and **P_3_**, which increases with the increasing of the molecular weight of polyether, was 13,510, 15,200, and 20,800 g/mol, respectively. Obviously, the number-average molecular weight of polymers is greater than those of the sum of POSS molecule and eight polyether segments. However, the difference between the number-average molecular weight of the three polymers (1690 and 5600 g/mol) is very consistent with the result of the octa-substituted polymer.

### 3.3. Self-Assembly Behaviour of Polymers

**Figure 4:**
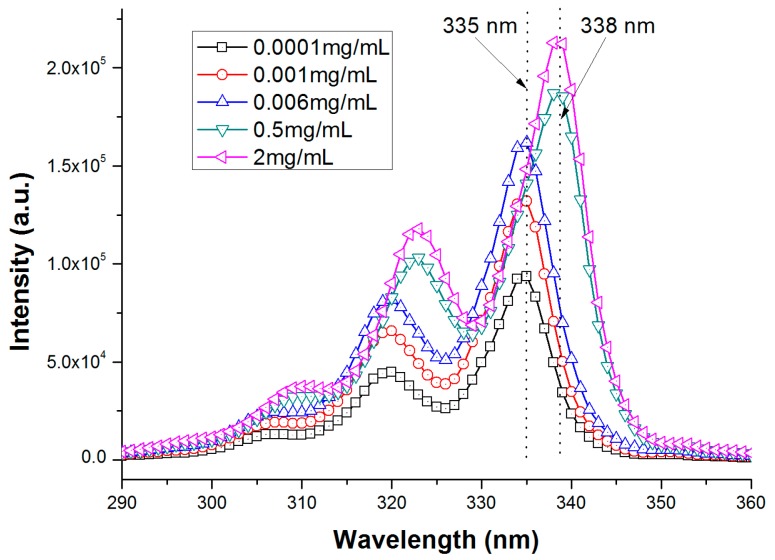
Excitation spectra of pyrene in aqueous solution of **P_1_** at various concentrations.

The micelle formation of the amphiphilic polymers was confirmed by fluorescence technique using pyrene as a probe. Figure 4 shows the fluorescence-excitation spectra of pyrene in POSS–PEG micelles at different concentrations. A red shift of the absorption peak from 335 to 338 nm was observed when the concentration of polymer was increased from 1.0 × 10^−4^ to 2.0 mg/mL. This red shift is attributed to the transfer of pyrene molecules from the hydrophilic environment to the hydrophobic micellar core. The results supply information on the location of the pyrene molecule in the system, that is, micelles are formed [53].

**Figure 5:**
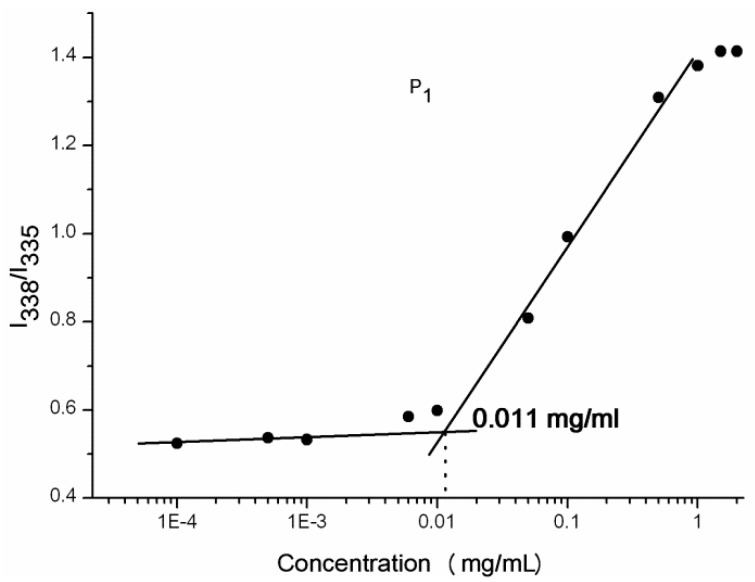
Variation of the intensity ratio (*I*_338_/*I*_335_) as a function of **P_1_** concentrations, the dotted line shows the CMC value.

CMC is one of the key parameters for studying the formation of micelles and evaluating the stability of the resulting micelles. The intensity ratio of *I*_338_/*I*_335_ versus log*C* of the polymers in aqueous solution is shown in Figure 5. From these plots, the CMC of **P_1_** was determined to be approximately 1.1 × 10^−2^ mg/mL through the intersection of two straight lines. The CMC of **P_2_** and **P_3_** were also obtained by the same method and shown in Figure S4. The CMC values of the three polymers increased as the proportion of polyether segment increased, which is consistent with the expected order (but it is different from polysiloxane system [54]). For polysiloxane system, because of the formation of vesicles and lamellae, the CMC values decreased as the proportion of polyether segment increased. Generally, amphiphilic polymers with higher content of the hydrophobic segments will cause stronger interactions between hydrophobic chains, leading to a more stable structure and, therefore, to a lower CMC value. 

**Figure 6:**
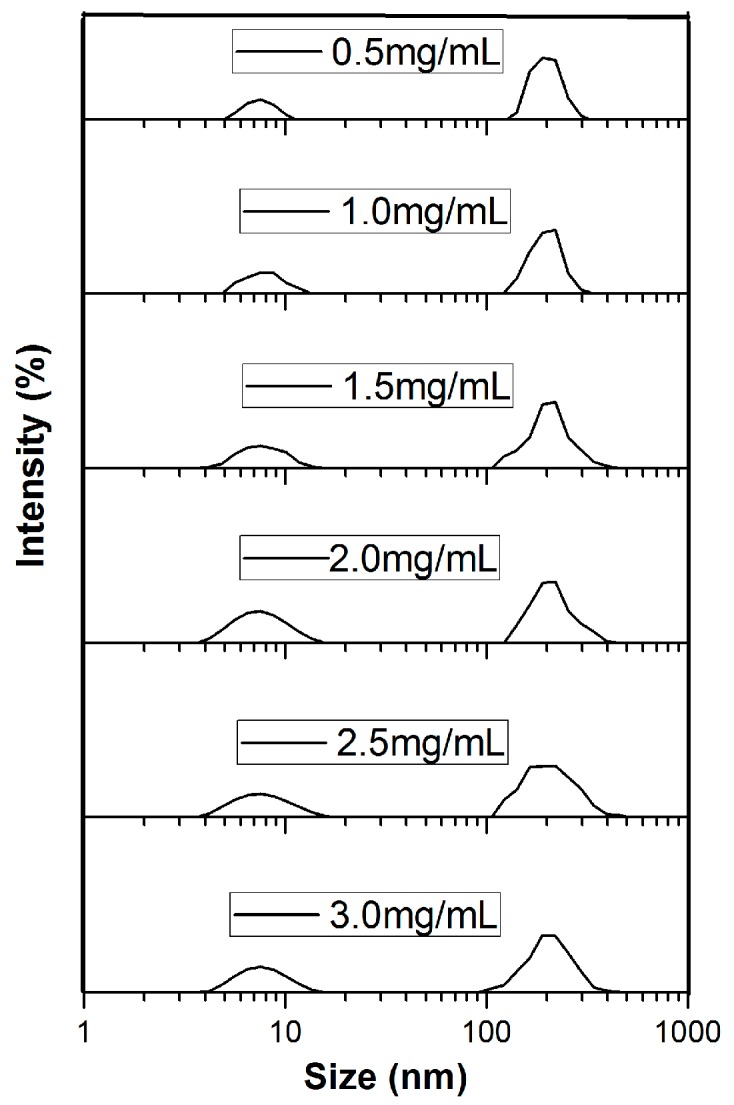
Size distribution of nanoparticles formed by different concentration of **P_1_** determined by DLS at 25 °C in aqueous solutions.

To study the self-assembly nanoparticle formation, the particle size was studied using dynamic light scattering, and the results are shown in Figure 6.

**Figure 7:**
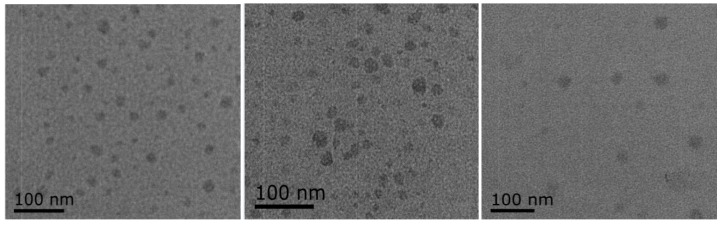
Typical TEM images of copolymers micelles (1 mg/mL), from left to right are **P_1_**, **P_2_**, and **P_3_**, respectively.

The DLS results showed that a bimodal (two peaks) distribution was observed at all concentrations. The small peak represents the unassociated unimolecular micelle with *R*_h_ = ~7 nm and the dominant peaks attributed to the scattering from the large particles attributable to the aggregation of POSS–PEG. The unassociated unimolecular micelle and aggregate micelle are in a state of dynamic balance. The aggregation peak is observed, even at a concentration of 0.5 mg/mL, suggesting that the CMC of POSS–PEG is lower than 0.5 mg/mL, which is in agreement with the fluorescence measurements (CMC = 0.011 mg/mL). Figure 6 shows that both of the micelle sizes in aqueous solution do not depend on the polymer concentration, indicating that the measurements were carried out in the dilute concentration regime and that the micelle formation followed a closed association mechanism [55]. This is also consistent with the previous literature [56]. The particle size obtained from TEM micrographs are smaller than those obtained by DLS (Figure 7). TEM micrographs show the micelle with the corona shrinkage after the evaporation of water, while DLS gives the size of the swollen nanoparticles in aqueous solutions. From the TEM images, there are two kinds of size of micelles in the three polymers; the small micelles are several nanometers and the large micelles are dozens of nanometers. At the same time, there is a slight difference of the morphological micelles between the three polymers. These results are consistent with the measurements of DLS.

### 3.4. Thermal Response Aggregation Behavior

POSS–PEG polymer has the temperature sensitivity because of the hydrogen bonding interaction with the water molecules, and the hydrogen bond is destroyed gradually with the increase of temperature. In particular, the polyether backbone containing structural units of propylene glycol becomes hydrophobic with the increment of temperature. Micelles formed by the POSS–PEG polymer are comprised of hydrophobic POSS as the core and a hydrophilic polyether chain as the outer shell, which optimizes the surface contacting with water and prevent the micelles to aggregate further. If this hydrophilic outer shell becomes less hydrophilic with the increase of temperature, the micelles will aggregate, then resulting in turbidity of solution. Thus, the polymer has a lower critical solution temperature (LCST). The thermo-sensitive behaviors of POSS–PEG nanoparticles were investigated by measuring the cloud point (CP). In order to ensure the formation of polymer nanoparticles, the concentrations of polymer aqueous solution were of 5 mg/mL.

**Figure 8:**
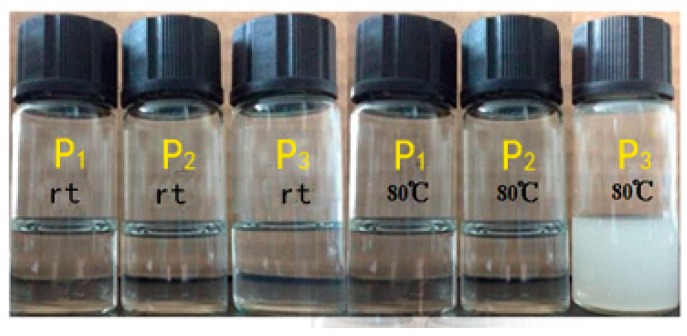
Digital photographs of transmittance variation of POSS–PEG aqueous solution (5 mg/mL).

**Figure 9:**
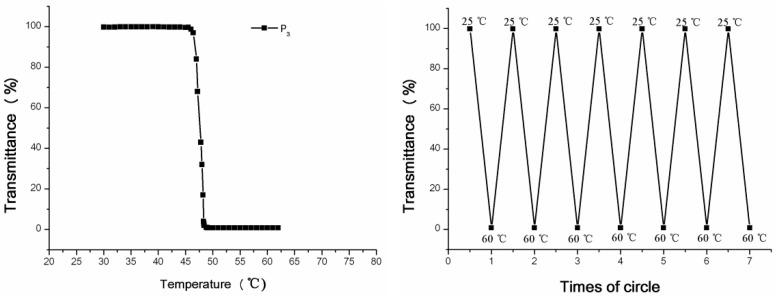
Temperature dependence of optical transmittance and reversible change of transmittance at 550 nm for **P_3_** aqueous solution (5 mg/mL).

The polymers **P_1_** and **P_2_** did not show a significant temperature response behaviour, they were colourless and transparent, while the **P_3_** changed from a colourless solution to turbid white (Figure 8). This is mainly because of the different composition of the polyether. In **P_1_** and **P_2_**, the EO/PO of the polyether chain were 70/30 and 85/15, in which the PO content dominated thermo-sensitive behaviours [57,58]. While in the polymer **P_3_**, the EO/PO was 40/60, thus **P_3_** had obvious temperature sensitivity. The transmittance curve of the polymer **P_3_** was shown in Figure 9. When the temperature was above CP, the transparent **P_3_** aqueous solution suddenly became turbid, caused by the aggregation of **P_3_** nanoparticles. **P_3_** aqueous solution was sensitive to temperature, and the range of the transition temperature was less than 2 °C. It is worth noting that the thermo-sensitive behaviour of **P_3_** nanoparticles was completely reversible. The transmittance of the **P_3_** nanoparticles was about 100% at room temperature, and it was about 0 when heated to 60 °C, and when cooled to room temperature, it was nearly 100% again. 

### 3.5. Thermal Stability Analysis

The thermodynamic behaviour of allyl-polyether and octa-substituted POSS in the nitrogen atmosphere was studied by TGA, and the results were shown in Figures S5 and S6, respectively. It could be found that the allyl-polyether was one step degradation, the initial decomposition temperature was from 270 to 310 °C, and the decomposition temperature increased with the increase of the molecular weight of polyether. These results were consistent with that reported results about oligomeric allyl-polyether (molecular weight ranges from 146 to 322 g/mol) [37]. The results showed that the high molecular weight allyl-polyether had good thermodynamic stability.

**Figure 10:**
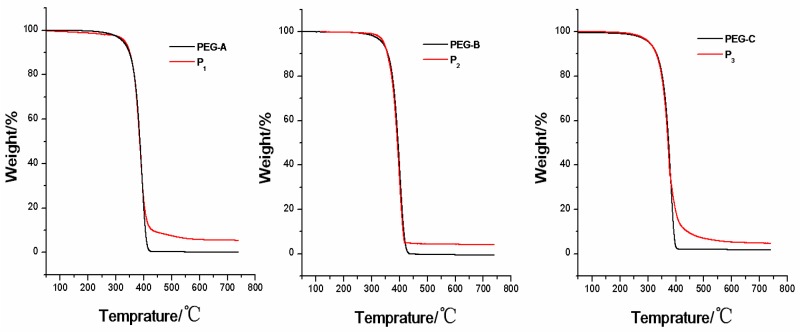
TGA curves of allyl-polyether and octa-substituted POSS (in N_2_).

The thermal decomposition temperature of the polymers were not much different from that of the corresponding polyether, which indicated the thioether bond formed by click reaction had no effect on the thermal stability of the polyether. The thermal analysis results of the small molecular weight polyether substituted POSS polymer showed that the thermal decomposition process was divided into two steps [37]. The first step was the polyether chain segment at 164–268 °C, and the second step was the POSS cage to SiO_2_ in the range of 359–394 °C. However, the current POSS–PEG polymers were different. Their curves of TGA had only one major degradation event similar to high molecular weight polyether, which might attribute to approximate decomposition temperature of high molecular weight polyether and the first decomposition temperature of POSS–SH (Figure 10). Although the second decomposition temperature of POSS–SH (decomposition temperature of POSS cage) presented about 400 °C (Figure S6), its decomposition process was not displayed in the TGA curves because the contents of POSS in polymers were very low. When the temperature was up to 600 °C, the residual amounts of three allyl-polyethers were close to almost zero, while those of polymer **P_1_**, **P_2_**, and **P_3_** were 5.5%, 4.0%, and 3.2%, respectively. The results showed that the contents of POSS in polymers were **P_1_** > **P_2_** > **P_3_**, which was consistent with the theoretical calculation results.

## 4. Conclusions

Three octa-substituted POSS amphiphilic polymers (**P_1_**, **P_2_**, and **P_3_**) were successfully prepared via a newly-designed thiol-ene click chemistry protocol. The results of ^1^H NMR, ^13^C NMR, ^29^Si NMR FT-IR, and gel permeation chromatography indicated the well-defined structures of the polymers. The experimental results showed that the amphiphilic polymers could form stable and bimodal nanoparticles directly by self-assembly in aqueous solution, and the formation of micelle followed closed association mechanism, in which the particle size was independent of the polymer concentration. Furthermore, the polymer **P_3_** showed obvious temperature responsive behaviour. When the temperature rose to 45.5 °C, the solution quickly transformed into a turbid white solution and the range of the transition temperature was less than 2 °C. Thermal analysis showed that the polymer had excellent thermal stability and the presence of the thioether bond did not affect its thermal stability. Due to the excellent properties of POSS, facile synthesis, amphipathy, direct self-assembly to form polymer nanoparticles, and thermo-sensitive behaviour, this kind of polymer would shine in many fields, especially in biomedical engineering.

## Supplementary Materials

The following are available online at www.mdpi.com/2073-4360/9/7/251/s1, Figure S1: The infrared spectrum of POSS–SH, Figure S2: ^1^H NMR spectra of POSS–SH, Figure S3: ^13^C NMR spectra (a) and ^29^Si NMR spectra (b) of POSS–SH, Figure S4: Variation of the intensity ratio (*I*_338_/*I*_335_) as a function of **P_2_** and **P_3_** concentrations, the dotted line shows the CMC value, Figure S5: TGA curves of PEG-A, PEG-B, and PEG-C (in N_2_), Figure S6: TGA curve of POSS–SH (in N_2_).
