# Supplementary material for: Facile Synthesis and Self-Assembly of Amphiphilic Polyether-Octafunctionalized Polyhedral Oligomeric Silsesquioxane via Thiol-Ene Click Reaction
Source: Polymers (Basel). 2017 Jun 28;9(7):251. doi: 10.3390/polym9070251 (PMC6432379; doi:10.3390/polym9070251)
Supplement: Supplementary file 1 [file polymers-09-00251-s001.pdf]

# Supplementary Materials: Facile synthesis and self-assembly of amphiphilic polyether-octafunctionalized polyhedral oligomeric silsesquioxane via thiol-ene click reaction

Yong Xia, Sha Ding, Yuejun Liu, Zhengjian Qi

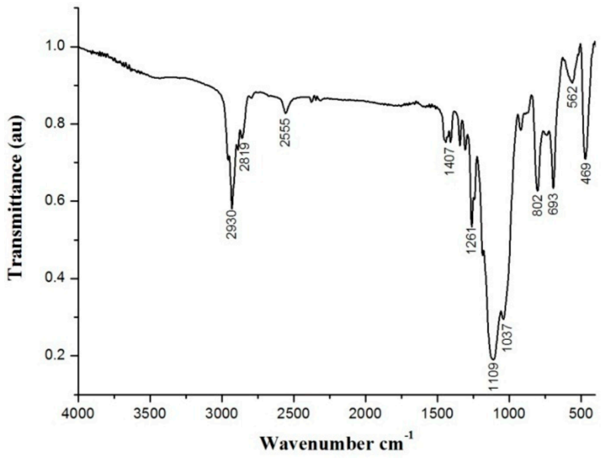


Figure S1 The infrared spectrum of POSS-SH

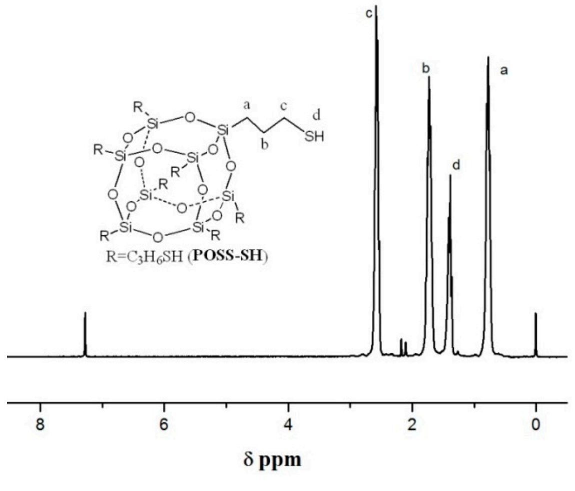


Figure S2 $^1$H NMR spectra of POSS-SH

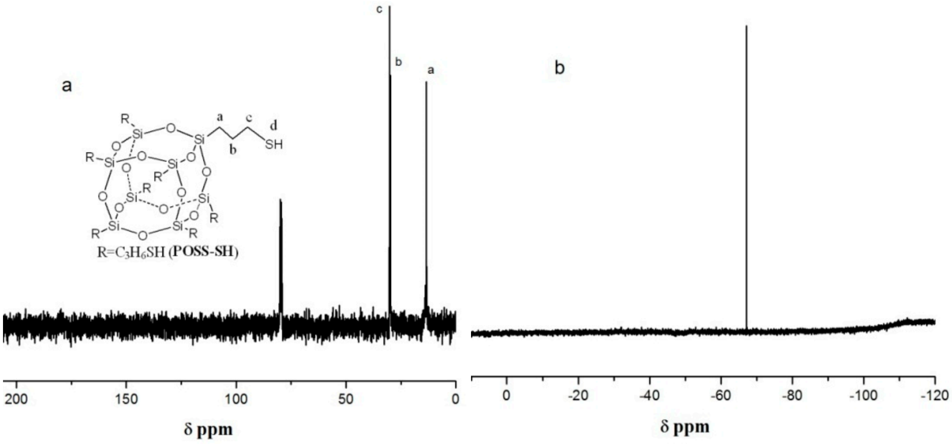


Figure S3 $^{13}C$ NMR spectra (a) and $^{29}Si$ NMR spectra (b) of POSS-SH

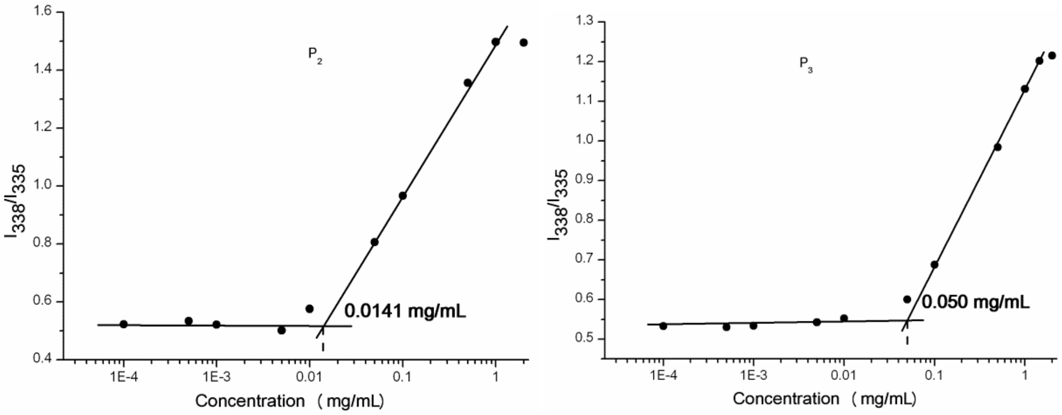


Figure S4 Variation of the intensity ratio ($I_{338}/I_{335}$) as a function of $\mathbf{P_2}$ and $\mathbf{P_3}$ concentrations, the dotted line shows the CMC value.

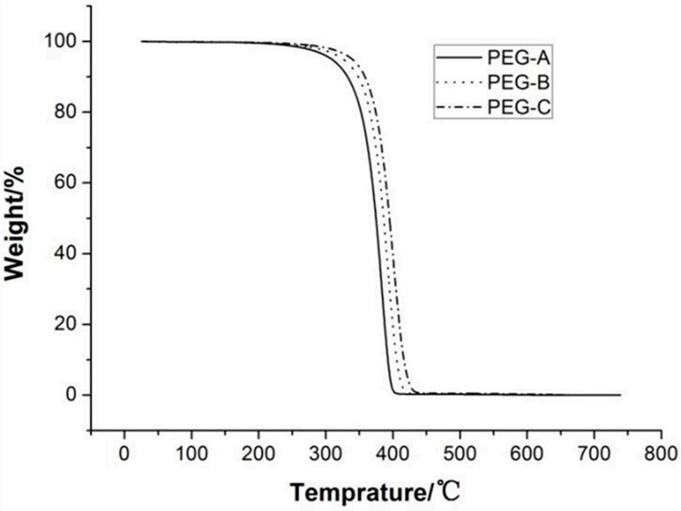


Figure S5 TGA curves of PEG-A, PEG-B, and PEG-C (in $N_2$)

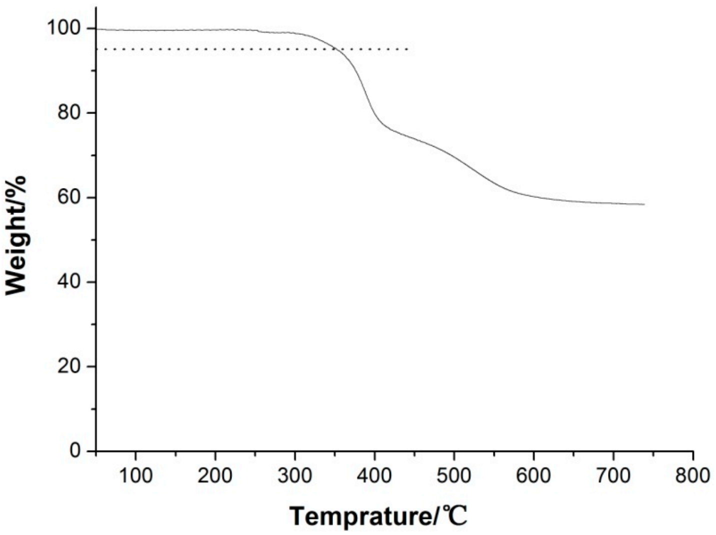


Figure S6 TGA curve of **POSS-SH** (in $N_2$)
